# Household Smoking Restrictions, Time to First Cigarette and Tobacco Dependence

**DOI:** 10.1155/2021/5517773

**Published:** 2021-04-15

**Authors:** Steven A. Branstetter, Nicolle Krebs, Joshua E. Muscat

**Affiliations:** ^1^Department of Biobehavioral Health, Pennsylvania State University, University Park State College, PA, USA; ^2^Penn State Cancer Institute, Pennsylvania State University, Hershey, PA, USA; ^3^Department of Public Health Science, Milton S. Hershey Medical School, Pennsylvania State University, University Park College State, PA, USA

## Abstract

**Objective:**

Environmental factors, such as household smoking restrictions (HSR), may impact a range of smoking-related outcomes. The current study examined the effects of various levels of HSR on smoking behaviors, including the number of cigarettes smoked per day and levels of nicotine dependence in a population of adult smokers. (1) Having specific HSR reduces the urges to smoke (path A); (2) having specific HSR reduces CPD (path B); (3) having specific HSR results in lower overall nicotine addiction (path C), and later, TTFC will be associated with (4) lower urges to smoke in the morning (path A'), (5) fewer CPD (path B'), and (6) lower levels of nicotine addiction (path C').

**Method:**

Regression models using self-reported data from the Pennsylvania Adult Smoking Study (*N* = 353) were used. TTFC was measured minutes between waking and the first cigarette of the day. Household smoking restrictions were measured as follows: (1) full ban on smoking in the home, (2) partial ban, or (3) no ban.

**Results:**

Subjects with no household smoking restrictions had lower incomes and education than those with at least some household smoking restrictions; those with full bans smoked less and had an earlier TTFC than those with at least some household smoking restrictions. Smokers with a full ban had a later TTFC, mediated by fewer cigarettes per day and lower cravings. Among those with partial bans, there is no reduction in cigarettes per day and an increase in urges to smoke.

**Conclusions:**

Partial household smoking restrictions are no better than no household smoking restrictions with regard to cigarettes per day and TTFC, and may cause an increase in urges to smoke in the morning.

## 1. Introduction

The time from waking in the morning until the first cigarette of the day is strongly associated with nicotine use behaviors and has increasingly been used as a single-item measure of dependence in a range of smoking studies, including clinical trials, laboratory studies, and other investigations of cigarette use [1–4]. The most commonly used time to first cigarette of the day (TTFC) item, “*how soon after you wake up do you smoke your first cigarette*?,” is derived from the Fagerstrom Test for Nicotine Dependence (FTND; [5]). This single item has been shown to be a robust predictor of smoking cessation outcomes, tobacco smoke toxicant exposure, risk of lung and head and neck cancers, and sleep outcomes, even when controlling for the number of cigarettes per day (CPD; [6–8]b; Mercincavage et al., In Press; [9]). Even among populations of adolescents and light- and nondaily smokers, TTFC remains a strong indicator of nicotine uptake, toxicant exposure, and outcomes ([8, 10]; Mercincavage et al., In Press).

Our understanding of TTFC as a physiological indicator of nicotine addiction may be changed if, in fact, environmental factors play a large role in driving this behavior. Indeed, the social influences of tobacco use are considerable and are impacted by proximity and interaction with other smokers [11]. Environmental and social controls of smoking behaviors, including self-imposed household restrictions or rules (HSR) on when or where smoking can take place within the home [12], may also influence patterns of tobacco use. Estimates suggest that nearly half of all households with at least one smoker may have some restrictions or rules on smoking in the home [13]. Research demonstrates that HSR are associated with higher intent to quit smoking [14], prolonged time to relapse after cessation [15], more successful cessation [16], reducing smoking initiation among adolescents [17], fewer CPD [18], and even better overall health than smokers with no household restrictions [19]. It is not yet clear if it is the presence of such rules that leads to improved outcomes or if individuals who are more motivated to quit, who are healthier, and who are lighter smokers are those more likely to implement these rules [18].

Regardless of the impetus for the creation of HSR, the presence of such rules may impact the measurement of TTFC and, consequently, TTFC's predictive validity on tobacco dependence. When examining the relation between HSR and TTFC, it is important to consider the contextual factors which may help resolve whether if TTFC is an independent measure of nicotine dependence or simply a correlate of environmental or social factors. For example, it is possible that the physical limitations placed on smokers in households with restrictions (e.g., getting out of bed, getting dressed, and moving to a designated smoking location) may push back the TTFC to a later category (e.g., from having the first cigarette within 5 minutes to having the first cigarette within 10 minutes). However, this may not affect the urges or cravings to smoke and may not affect the number of cigarettes smoked per day. The nature of the HSR is another important element to consider, as not all HSR are full bans on smoking in the home. Some households have partial bans, where smoking is allowed in some areas of the home but not in others. Others may have bans on combustible cigarettes, but not electronic nicotine delivery systems (e.g., e-cigarettes and JUUL). These partial bans may not impact the TTFC, if such an association exists, in a manner similar to a full ban.

Few studies have examined the impact of HSR on nicotine dependence [15, 16, 18, 20], and only a single study has examined the association between HSR and TTFC, using a sample of treatment-seeking smokers and nontreatment seeking smokers with serious mental illness. Steinberg and colleagues found that those with no HSR were significantly more likely to be in a lower TTFC category (i.e., smoke sooner after waking) compared to those with partial or full in-home smoking bans [21]. The present study sought to determine whether HSR predicts TTFC and other smoking-related behaviors in a sample of adult regular smokers not seeking to quit.

## 2. Methods

### 2.1. Participants and Procedures

Data for this study come from the Pennsylvania Adult Smoking Study (PASS), a study of nicotine dependence and smoking behaviors conducted between 2012 and 2014 [22]. Methods and details on the larger PASS study have been published elsewhere [23–25]. Inclusion criteria included being aged 18 or older, not immediately seeking to quit smoking, and smoking one or more cigarettes per day. Females who were pregnant were excluded. The study was approved by the Internal Review Board at Pennsylvania State University, and participants provided informed consent prior to participation. A total of 352 participants completed screening and signed the consent; one participant did not complete the full study protocol. After completing an eligibility screen, participants completed a 2-hour in-home administration of questionnaires and a preliminary interview.

### 2.2. Measures

#### 2.2.1. Demographic Information

Participants provided information on age, gender, race/ethnicity, educational attainment, marital status, and household income.

#### 2.2.2. Tobacco Use History

Participants reported on their tobacco use history by responding to items from the Consensus Measures of Phenotypes and Exposures (PhenX) toolkit (version 5.1, March 23, 2012). Items include age started smoking, number of cigarettes per day, and nicotine dependence as measured by the Fagerstrom Test for Nicotine Dependence (FTND; [5]) and the Hooked on Nicotine Checklist (HONC; [26]), two standard, well-validated measures of nicotine dependence [27]. TTFC was measured in actual minutes from the time of waking until the first cigarette of the day. For initial ANOVA analyses, TTFC was coded into one of four categories such as those used on the FTND (e.g., 0-5 minutes; 6-30 minutes; 31-60 minutes; >61 minutes). For linear regression analysis, TTFC was modeled using the reported minutes from waking until the first cigarette.

#### 2.2.3. Household Rules and Household Characteristics

Participants indicated the number of people living in their home and how many underage children (<18 years) lived in the home. Participants also indicated if they had rules regarding smoking in the household. Household smoking rules (HSR) were characterized as follows: (1) full ban, smoking not allowed anywhere in the home; (2) partial ban, smoking is allowed some places or sometimes within the home; and (3) no ban, smoking is allowed anywhere in the home/there are no “rules” regarding smoking in the home.

### 2.3. Analytic Strategy

All analyses were completed using SPSS, Version 25 (IBM Corp, Armonk, NY). Initial bivariate analysis includes Chi-square tests comparing HSR categories with TTFC categories. ANOVA with post hoc comparisons were conducted to determine differences in demographic and smoking behavior measure by HSR category. Linear regression analysis was conducted to determine predictors of TTFC, followed by multiple mediation analyses to examine plausible relations between variables.

**Figure 1:**
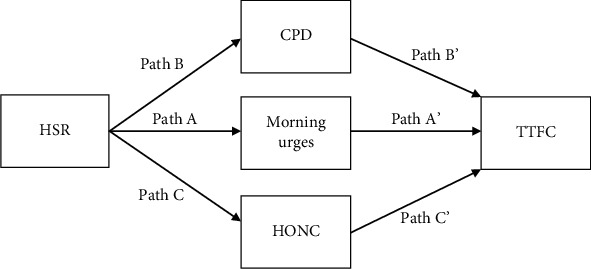
Multiple mediation model.

**Figure 2:**
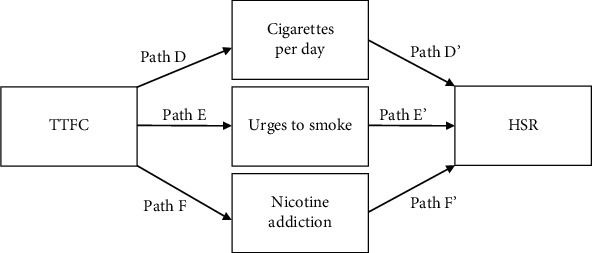
Alternative multiple mediation model.

**Figure 3:**
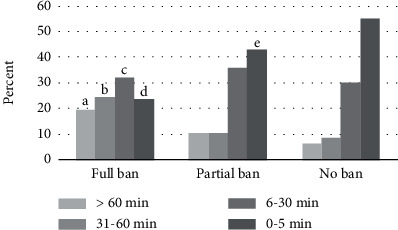
Chi-square differences between TTFC and household smoking restriction. Note: a, b, d: significantly different than both partial and no ban; c: not significantly different than partial or no ban; e: significantly different than no ban. All proportional differences at the.05 level.

We used multiple mediation analyses and bootstrapping methods with bias-corrected confidence intervals for all pathway models [28]. These models determined the effect of HSR on TTFC, exploring the potential simultaneous mediation of this relation by urges to smoke in the morning, average number of cigarettes per day, and nicotine addiction/loss of autonomy over smoking as measured by the HONC. The overall FTND scores were not used as a measure of addiction in models to avoid multicollinearity as two items from the scale (cigarettes per day and TTFC) are independently examined in the models. Mediation models examined the hypotheses that HSR may have the effects of as follows: (1) reducing the urges to smoke, perhaps as a result of the “hassle” associated with smoking behaviors in homes with HSR (path A in Figure 1), (2) reducing CPD, again this may be an effect of the additional behaviors required to smoke in a home with smoking restrictions in place (path B in Figure 1), (3) reducing the overall tobacco dependence/loss of autonomy over smoking associated with HSR (path C in Figure 1), and that a later TTFC is the result of (4) lower urges to smoke in the morning (path A' in Figure 1), (5) fewer CPD (path B' in Figure 1), and (6) lower levels of nicotine addiction/loss of autonomy (path C' in Figure 1).

The data used in the current analyses are cross-sectional, thereby limiting the interpretation of causality for the mediation analysis. It may be that implementing HSR affects smoking behaviors such as CPD and levels of addiction. Alternatively, smokers who smoke relatively fewer cigarettes or are less addicted may be more likely to implement HSR. Thus, we tested an alternate model to examine the model fit predicting HSR from TTFC, urges to smoke, and cigarettes per day, (Figure 2). By reversing the proposed predictors and outcomes, we can explore the potential pathways in more detail. Both the initial and alternative models reflect plausible associations between variables.

The models controlled for age (linear), gender (categorical), education (linear), total family income (linear), marital status (categorical), number of underage children in the home (linear), total number of people living in the home (linear), and age started smoking regularly (linear). Household smoking rules were dummy coded as (1) full ban vs. partial or no ban, (2) partial ban vs. full or no ban, and (3) no ban vs. partial or full ban. Given that multiple models were conducted, we controlled for potential Type II errors using false discover rate (FDR) corrections of *p* values [29]; all reported *p* values are the adjusted FDR values.

## 3. Results

### 3.1. Description of the Sample

All data were initially screened to insure they met assumptions of normality of distribution and to examine for patterns of missingness. TTFC and total family income demonstrated significantly skewed values and were therefore normalized using log transformations. Log-transformed values were used in the analyses whereas actual, pretransformed values are reported in descriptive tables for interpretability. No variables were missing more than 5% of the data, and therefore, no further adjustments were made [30]. Overall, the sample was 42.8% male (*N* = 151); they were an average of 36.88 (SD = 11.74) years old and were 87% white, 9.1% African American, and 4% other. The full sample smoked an average of 16.5 (SD = 8.1) cigarettes per day and smoked an average of 25.17 (SD = 26.79) minutes after waking. A total of 159 (45%) came from households with full smoking bans, 112 (31.9%) came from households with partial smoking bans, and 80 (22.8%) came from households with no rules about smoking in the home (see Table 1 for full description of the sample).

### 3.2. Household Smoking Rules and Smoking Behaviors

One-way ANOVA with Scheffe's post hoc analyses examined differences on demographic and smoking behavior measure by HSR category (see Table 1). Overall, those households with full smoking bans were significantly younger than those with either partial bans or no bans, *F*(2, 346) = 21.37, *p* < .001. Those with no HSR had lower levels of educational attainment than those with full smoking bans, *F*(2, 348) = 6.5, *p* = .002, and those with no HSR had lower overall income than those with partial or full smoking bans, *F*(2, 347) = 7.7, *p* = .001. Individuals with full smoking bans had more children in the household than those with no HSR, *F*(2, 348) = 3.89, *p* = .02; there were no difference in the number of underage children between those with no bans and those with partial bans. With regard to smoking behaviors, those with full smoking bans smoked fewer cigarettes per day than those with either partial or no HSR, *F*(2, 348) = 9.8, *p* < .001; there were no differences in cigarettes per day between those from partial and no ban households. Likewise, those with a full ban had a later TTFC than those with partial or no HSR, *F*(2, 348) = 19.16, *p* < .001, with no differences between those with partial and no bans. Individuals with full smoking bans had lower urges to smoke in the morning than those with partial or no smoking bans, *F*(2, 348) = 9.28, *p* < .001; again, there were no differences between partial and no-bans households. Chi-square analyses determined there were no significant differences between HSR categories and marital status or gender.

Overall, with regard to social, demographic, and environmental contexts, those with full smoking bans are younger, are more educated, have greater household incomes, and have more underage children in the home than those with no smoking restrictions. There were no differences between those with full and partial smoking bans on household income, education, and the number of children in the home; however, those with partial bans were older than those with full bans. With regard to smoking behaviors, those with full smoking bans smoked fewer cigarettes per day, had later TTFC, and had lower urges to smoke than both those with partial and no smoking bans.

### 3.3. Household Smoking Rules and Time to the First Cigarette of the Day

For purposes of comparison to literature using categorical measures of TTFC, Chi-square analyses examined HSR category (full, partial, or no smoking ban) and TTFC category (e.g., smoking 0-5 minutes; 6-30 minutes; 31-60 minutes; >61 minutes after waking). Overall, the model highlighted differences between groups, *χ*^2^(6, *N* = 351) = 35.17, *p* < .001, with those with full bans being more likely to smoke between 31 and more than 60 minutes after waking than those with partial or no smoking bans. Those with no bans were more likely to smoke within 5 minutes of waking than those with either full or partial bans. However, there were no differences between groups on smoking between 6 and 30 minutes of waking (see Table 2 and Figure 3).

Initial linear regression models examined if having full household smoking bans, versus partial bans and no restrictions on smoking, was predictive of TTFC controlling for age, gender, educational attainment, household income, average cigarettes per day, and loss of autonomy as measured by the HONC. For these models, TTFC was used as a continuous variable of actual minutes between waking and the first cigarette of the day. The full model demonstrated good fit, *F*(7, 342) = 22.73, *p* < .001, adjusted *R*^2^ = .31. Findings demonstrated that education, *β* = .12, *t* = 2.76, *p* = .006, cigarettes per day, *β* = −.33, *t* = −.6.90, 2.76, *p* < .001, nicotine addiction (honc), *β* = −.23, *t* = −4.74, *p* < .001, and full household smoking bans, *β* = .15, *t* = 3.03, *p* = .002, all predicted TTFC.

Next, models examined if having only partial smoking bans, versus full bans and no smoking restrictions, predicted TTFC. The overall model fit was adequate, *F*(7, 342) = 21.42, *p* < .001, adjusted *R*^2^ = .29. Findings demonstrated that age, *β* = −.11, *t* = −2.42, *p* = .02, education, *β* = .14, *t* = 3.08, *p* = .002, cigarettes per day, *β* = −.35, *t* = −.7.23, 2.76, *p* < .001, and nicotine addiction, *β* = −.22, *t* = −4.66, *p* < .001, all predicted TTFC. However, partial smoking bans did not predict TTFC, *β* = −.08, *t* = −1.72, *p* = .08.

Final models examined if having no smoking bans, versus full or partial bans, predicted TTFC. The overall model fit was adequate, *F*(7, 342) = 21.34, *p* < .001, adjusted *R*^2^ = .29. In the final model, education, *β* = .13, *t* = 2.84, *p* = .005, cigarettes per day, *β* = −.35, *t* = −7.13, *p* < .001, and nicotine addiction, *β* = −.23, *t* = −4.80, *p* < .001, all predicted TTFC. Having no household rules regarding smoking did not predict TTFC, *β* = −.08, *t* = −1.61, *p* = .11.

Alternative multinomial regression models were conducted reversing the order of the predictor and criterion (HSR and YYFC); these models examined if TTFC could predict HSR group (full, partial, or no ban) controlling for age, gender, educational attainment, household income, underage children in the home, number of people living in the home, average cigarettes per day, and HONC scores. Results demonstrate adequate model fit, *χ*^2^(22) = 83.35, *p* = <.001, Cox and Snell pseudo − *R*^2^ = .25. In the model, compared to those with full smoking ban, no smoking bans were associated with earlier TTFC, *β* = −1.31 (SE = .44), Wald = 8.60, *p* = .003, older age, *β* = .06 (SE = .01), Wald = 16.99, *p* < .001, and lower total household income, *β* = 1.26 (SE = .52), Wald = 5.69, *p* = .02. Compared to those with partial smoking bans, no smoking bans were associated with being older, *β* = −.05 (SE = .02), Wald = 9.13, *p* = .003, and having lower incomes, *β* = 1.43 (SE = .56), Wald = 6.49, *p* = .01. TTFC did not differentiate between those with partial or no smoking bans, *β* = .74 (SE = .43), Wald = 2.96, *p* = .09. Finally, compared to full smoking bans, partial smoking bans were associated with earlier TTFC, *β* = −.80 (SE = .35), Wald = 5.13, *p* = .02.

Overall, as expected, CPD and nicotine addiction as measured by the HONC predicted TTFC in regression models. Additionally, education emerged as a significant predictor or TTFC for those in all HSR categories. Alternate models predicting HSR categories found that age and income differentiated those with no bans from those with full or partial bans whereas an earlier TTFC differentiated between those with full bans from those with no or partial bans.

### 3.4. The Effects of Household Smoking Rules on Time to the First Cigarettes of the Day

Multiple mediation models were guided by six hypotheses (see Figure 1): (1) having specific HSR reduces the urges to smoke (path A in Figure 1); (2) having specific HSR reduces CPD (path B); (3) having specific HSR is associated with lower overall nicotine addiction (path C); and later, TTFC will be associated with (4) lower urges to smoke in the morning (path A'), (5) fewer CPD (path B'), and (6) lower levels of nicotine addiction (path C').

Findings of the first model examining the effect of a full household ban suggest an overall appropriate model fit, *F*(12, 281) = 27.26, *p* < .001, adjusted *R*^2^ = .52. Having a full smoking ban was associated with a reduction in urges to smoke in the morning, *β* = −.87 (SE = .29), *t* = −3.01, *p* = .002 (path A), and a reduction in cigarettes per day, *β* = −2.41 (SE = .89), *t* = −2.73, *p* = .01 (path B), but not with a reduction in nicotine addiction as measured by the HONC, *β* = −.25 (SE = .23), *t* = −1.05, *p* = .30 (path C). Urges to smoke was associated with TTFC, *β* = −4.46 (SE = .45), *t* = −9.88, *p* < .001 (path A'), as were cigarettes per day, *β* = −.81 (SE = .14), *t* = −5.68, *p* < .001 (path B'). The HONC was not associated with TTFC, *β* = −.99 (SE = .56), *t* = −1.78, *p* = .08 (path C'). There was a direct effect of a full household smoking ban on TTFC, independent of the mediators, *β* = .13 (SE = .05), *t* = 2.04, *p* = .04. There was an indirect effect of HSR on TTFC mediated through CPD, *β* = .05 (SE = 02), 95% CI: .01–.009, and through urges to smoke, *β* = .09 (SE = .03), 95% CI: -.10–-.0002. In this and all multiple mediation models that follow, the covariate “age at which participants started smoking” was associated with an earlier TTFC, *β* = .01 (SE = .005), *t* = 2.68, *p* = .007 (see Table 3 for full multiple mediation results).

The next model examined the effect of a partial smoking ban and demonstrated an acceptable model fit, *F*(12, 281) = 26.15, *p* < .001, adjusted *R*^2^ = .51. Interestingly, in this model, a partial smoking ban had the effect of increasing urges to smoke in the morning, *β* = .91 (SE = .33), *t* = 2.78, *p* = .005 (path A), but had no effect on either cigarettes per day, *β* = 1.25 (SE = 1.00), *t* = 1.23, *p* = .21 (path B) or HONC scores, *β* = .42 (SE = .26), *t* = −1.66, *p* = .10 (path C). Urges to smoke in the morning, *β* = −.10 (SE = .01), *t* = −10.49, *p* < .001 (path A'), and cigarette per day, *β* = −.84 (SE = .14), *t* = 5.89, *p* < .001 (path B'), were associated with TTFC; however, HONC scores, *β* = −.97 (SE = .56), *t* = −1.72, *p* = .08 (path C'), were not. There was no direct effect of a partial household smoking ban on TTFC, independent of the mediators and control variables, *β* = −1.45 (SE = 2.29), -.63, *p* = .52. There was an indirect effect of HSR on TTFC mediated through urges to smoke, *β* = −.10 (SE = .04) 95% CI: -.10–-.03.

The final multiple mediation models examined the effect of having no household smoking ban on TTFC. The overall model fit was acceptable, *F*(12, 281) = 27.04, *p* < .001, adjusted *R*^2^ = .52. In these models, having no smoking ban was associated with more average cigarettes per day, *β* = 2.40 (SE = 1.18), *t* = 2.05, *p* = .04 (path B), but had no effect on either urges to smoke in the morning, *β* = −.003 (SE = .39), *t* = −.009, *p* = .99 (path A), or HONC scores, *β* = −.36 (SE = .30), *t* = −1.21, *p* = .22 (path C). In this model, urges, *β* = −.77 (SE = .01), *t* = −10.74, *p* < .001 (path A'), and cigarettes per day, *β* = −.02 (SE = .003), *t* = −5.75, *p* < .001 (path B'), were both associated with TTFC; however, HONC was not, *β* = −.01 (SE = .01), *t* = −.90, *p* = .36. There was a direct effect of no smoking bans on an earlier TTFC, *β* = .14 (SE = .06), *t* = −2.33, *p* = .02. There was an indirect effect of HSR on TTFC mediate through cigarettes per day, *β* = −.05 (SE = .03) 95% CI: -.10–-.0002.

Overall, full household bans have both a direct effect on TTFC and indirect effects mediated through cigarettes per day and urges to smoke. No household bans had a direct effect and an indirect effect, mediated through cigarettes per day, on TTFC. Having a partial ban had no direct effect on TTFC but did have an indirect effect, mediated through urges to smoke.

### 3.5. Alternative Model

In this alternative model with the predictor and outcome variables (HSR and TTFC) reversed, the hypotheses are (1) later TTFC is associated with fewer cigarettes per day (path D in Figure 2), (2) later TTFC is associated with fewer urges to smoke in the morning (path E), (3) later TTFC is associated with lower levels of nicotine addiction (path F), (4) fewer cigarettes per day is associated with a greater likelihood of implementing any (full or partial) HSR (path D'), (5) lower urges to smoke is associated with a greater likelihood of implementing HSR (path E'), and (6) lower levels of nicotine addiction is associated with a greater likelihood of implementing HSR (path F').

Findings of the alternative analyses found later TTFC are significantly associated with (1) fewer cigarettes per day, *β* = −6.52 (SE = .72), *t* = −9.03, *p* = <.001 (path D), (2) fewer urges to smoke, *β* = −2.84 (SE = .21), *t* = −13.57, *p* < .001 (path E), and (3) less nicotine addiction, *β* = −1.30 (SE = .19), *t* = −6.63, *p* < .001 (path F). Finally, TTFC did have a significant direct effect on the likelihood of having HSR in the home, *β* = .87 (SE = .33), *z* = 2.61, *p* = .009 (results are expressed in log-odds metric), cigarettes per day, *β* = .03 (SE = .02), *z* = 1.47, *p* = .14 (path D'), urges to smoke, *β* = −.03 (SE = .06), *z* = −.49, *p* = .62 (path E'), and loss of autonomy over smoking, *β* = .09 (SE = .07), *z* = 1.20, *p* = .23 (path F'), which did not have significant associations with HSR in these models. There were no significant indirect (mediated) effects of TTFC on HSR in this model.

## 4. Discussion

The findings of the present study demonstrate a complex relation between household smoking restrictions and the time to the first cigarette of the day. We found that a full smoking ban was more likely to be categorized in the 31-60-minute and more than 60-minute categories of TTFC than those with partial or no bans, and less likely to be in the 0-5-minute category. Having a full smoking ban and having no smoking ban were both directly related to TTFC, even when considering smoking-related behaviors such as cigarettes per day and other social and demographic factors such as education, income, and age; however, there was no direct effect of having only a partial ban on TTFC. There were also indirect effects and mediated effects of HSR on TTFC, and these mediated effects varied by HSR category: for those with a full smoking ban, both cigarettes per day and urges to smoke were mediators, for those with no smoking bans only cigarettes per day mediated the effect, and for those with partial bans, only urges to smoke mediated the relation between HSR and TTFC. Additionally, findings demonstrate that those with partial smoking bans tend to be similar to those with full smoking bans with regard to social and demographic factors such as education and income but were more similar to those with no bans on smoking-related factors such as cigarettes per day, TTFC, and urges to smoke.

The time to first cigarette is often considered the best single marker of nicotine addiction, in part because it is also the best behavioral indicator of nicotine intake and nicotine biomarkers, and because it is relatively easy to assess. The utility of a highly sensitive and specific single indicator has advanced the understanding of nicotine addiction; however, there remains a lack of understanding as to how social environmental factors and physiologic measures of addiction help predict TTFC, and the interrelationships of these behaviors to TTFC. One important physiological measure in particular is smoking urges or cravings, which are considered an essential underlying characteristic of nicotine dependence, and which may, in turn, be affected by environmental factors that prompt smoking urges^29^. In the current study, we show that a full household smoking ban is associated with a reduction in CPD and urges to smoke in the morning; both of which are associated with an increased TTFC. However, partial smoking bans were not associated with the number of cigarettes per day and were associated with increased urges to smoke.

These findings help to understand why TTFC is a strong predictor of dependence; namely, that it is partially driven by cravings/urges, a defining characteristic of dependence and may be further influenced by HSR. For example, the relation between HSR and TTFC may come through the effect a smoking ban has on removing or altering smoking cues, which trigger an urge to smoke^30^. For example, when a smoker awakes, if a pack of cigarettes is not adjacent to the bed, the immediate urge to smoke may be reduced. Nevertheless, the specific aspects of home restrictions that may affect smoking behaviors needs further investigation; it may be that the relation between home restrictions and TTFC may reflect efforts to reduce overall smoking by lighter, less-addicted, smokers who are more motivated to quit. Whereas conventional wisdom might suggest that household restrictions may reflect motivation to reduce secondhand smoke for others in the household (e.g., children and other individuals in the household); however, findings of the current study show that, whereas individuals with full smoking bans had more underage children in the home, the presence of underage children or the number of other individuals in the household was not a significant covariate in any of the models examining the relation between HSR, cigarettes per day, nicotine addiction, and TTFC.

In addition to the above factors, the current study allowed us to determine the association of other salient behaviors with nicotine dependence that may be associated with TTFC, as indicated by alternative measures of dependence. For example, full or partial bans had no effect on the HONC score in mediation analysis. The HONC is a measure of the loss of autonomy, a theory of nicotine dependence that considers different behavioral measure of nicotine dependence than physiological nicotine cravings that precede the desire to smoke. Thus, whereas home smoking restrictions may have some effect on key behaviors associated with nicotine dependence, such as the number of cigarettes per day, it did not have an effect on this dimension of dependence.

Importantly, the findings of the current study demonstrated that the effects of household smoking restrictions on TTFC depended on whether the household restriction was a full or partial ban. Overall, it was found that individuals with partial smoking bans appear to be more similar to those who implement full smoking bans with regard to education and income yet are more similar to those who have no household smoking rules at all when it comes to nicotine dependence as measured by TTFC and CPD. Not only did partial household smoking bans not have an impact on TTFC or CPD, partial bans were associated with an increase in cravings. Whereas the study was not able to assess if partial bans are effective at decreasing secondhand smoke in the home for others living in the home, it does demonstrate that household rules that do not include a full ban on smoking in the home are not associated with changes in nicotine dependence, CPD, and may increase urges to smoke.

This study should be evaluated in light of its limitations. The nature of the cross-sectional does not allow for casual inference; thus, all models in the current study are presented as plausible associations between these variables which may inform future studies. We presented an alternative model in which the independent and dependent variables were switched in the sequence of relations. In the alternative model, TTFC had a direct effect on HSR, but there were no significant mediational pathways. However, regardless of the model, there is a clear association between TTFC and HSR. The present study offers potential mechanisms for this relation; however, further research is warranted to understand how HSR may impact the measurement of TTFC, whether or not TTFC should be “adjusted” or weighted based on the presence of HSR, and specifically, how HSR may be the result or cause of altered smoking behaviors. Additionally, the definition of “partial” smoking ban was somewhat ambiguous, and we could not quantify how much, when, and where smokers could smoke in the home—or if these bans were enforced, by either the participant or others in the home. It is possible that some participants who said they lived under a partial smoking ban may have had no effective restrictions on their in-home smoking.

## Figures and Tables

**Table 1:** Sample descriptive statistics.

	Full smoking ban			Partial smoking ban			No smoking ban		
	Male	Female	Total	Male	Female	Total	Male	Female	Total
Age	33.7 (10.2)	35.1 (10.4)	34.5^∗^ (10.3	38.1 (11.7)	38.1 (11.1)	38.1 (11.2)	43.3 (11.3)	44.5 (12.5)	44.2 (12.1)
Education	15.4 (1.9)	15.9 (2.1)	15.6 (2.1)	15.2 (2.1)	15.3 (1.2)	15.3 (1.2)	14.7 (1.9)	14.6 (2.1)	14.6 ^**‡**^ (2.0)
Household income	64k (38k)	60k (43k)	62k (41k)	61k (42k)	54k (29k)	57k (36k)	45k (28k)	42k (30k)	43k ^**‡**^ (30k)
Underage children	1.2 (1.3)	.98 (1.2)	1.1 **†** (1.2)	.78 (.95)	1.1 (.94)	.93 (.95)	.76 (1.1)	.60 (.84)	.70 (.94)
Urges to smoke	4.86 (2.53)	5.60 (2.97)	5.28 ^∗^ (2.80)	6.41 (2.57)	6.53 (2.08)	6.50 (2.32)	6.77 (2.50)	6.25 (2.58)	6.44 (2.55)
No. of people in household	3.6 (1.5)	3.2 (1.4)	3.3 (1.5)	3.2 (1.5)	3.3 (1.1)	3.3 (1.3)	2.7 (1.6)	3.1 (1.8)	3.0 (1.7)
Cigarettes per day	14.7 (7.4)	14.5 (7.9)	14.6^∗^ (7.7)	18.8 (8.1)	16.2 (7.1)	17.5 (7.7)	21.3 (9.0)	18.0 (8.4)	19.1 (8.7)
Time to first cigarette	32.8 (23.1)	33.6 (32.3)	33.2^∗^ (23.1)	17.5 (21.2)	22.7 (27.1)	20.2 (24.5)	12.3 (14.2)	17.0 (22.9)	15.3 (20)
HONC score	6.6 (2.4)	7.4 (2.1)	7.1 (2.2)	7.2 (2.2)	7.7 (1.8)	7.4 (2.0)	7.0 (2.0)	7.6 (1.9)	7.4 (2.0)
Age started smoking	17.1 (4.1)	16.9 (4.4)	16.9 (4.3)	16.9 (4.8)	16.6 (3.7)	16.8 (4.3)	17.4 (6.5)	16.1 (4.8)	16.5 (5.5)
Race									
White	87%	91.1%	89.3%	88.7%	88.1%	88.4%	82.8%	78.4%	80%
Black	8.7%	5.6%	6.9%	7.5%	6.8%	7.1%	13.8%	17.6%	16.3%
Other	4.3%	3.3%	3.8%	3.8%	5.1%	4.5%	3.4%	3.9%	3.8%
Hispanic	2.9%	2.2%	2.5%	1.9%	3.4%	2.7%	3.4%	2.0%	2.5%

Note: ^∗^full smoking ban significantly different than partial smoking ban and no smoking ban groups, *p* < .01; ^‡^no smoking ban significantly different than full smoking band and partial smoking ban groups, *p* < .01; ^†^full smoking ban significantly different than the no smoking ban group, *p* < .01. Income rounded up for presentation in table format.

**Table 2:** Chi-square of TTFC and HSR category.

		>60 min	31-60 min	6-30 min	0-5 min	Total
Full smoking ban	Count	31	39	51	38	159
	Percent	19.5%	24.5%	32.1%	23.9%	100.0%
Partial smoking ban	Count	12	12	40	48	112
	Percent	10.7%	10.7%	35.7%	42.9%	100.0%
No smoking ban	Count	5	7	24	44	80
	Percent	6.3%	8.8%	30.0%	55.0%	100.0%
Total	Count	48	58	115	130	351
	Percent	13.7%	16.5%	32.8%	37.0%	100.0%

**Table 3:** Multiple mediation results.

	Full smoking ban				Partial smoking ban				No smoking ban			
	*β*	SE	*t*	*p*	*β*	SE	*t*	*p*	*β*	SE	*t*	*p*
HSR => CPD	-2.41	.89	-2.7	.01	1.25	.99	1.23	.21	2.40	1.18	2.05	.04
HSR => urges	-.87	.29	-3.01	.002	.91	.33	2.78	.005	-.003	.39	-.009	.99
HSR => HONC	-.25	.23	-1.05	.30	.42	.26	1.66	.10	-.36	.30	-1.21	.22
CPD => TTFC	-.81	.14	-5.68	<.001	-.84	.14	-5.89	<.001	-.01	.003	-5.75	<.001
Urges => TTFC	-4.46	.45	-9.88	<.001	-.10	.01	-10.5	<.001	-.11	.01	-10.7	<.001
HONC => TTFC	-.99	.56	-1.78	.08	-.97	.56	-1.72	.08	-.01	.01	-.89	.36
HSR => TTFC (direct effect)	.13	.05	2.04	.04	-1.45	2.29	-.63	.52	-.14	.06	-2.33	.02

Note: => indicates pathway between variables (see Figure 1). HSR: household smoking rules; CPD: average cigarettes per day; HONC: hooked on nicotine checklist; TTFC: time to first cigarettes.

## Data Availability

Data is available upon request of the authors.

## Additional Points

*Public Health Statement*. Smokers regularly self-impose various types of restrictions on where they can smoke within their homes; however, anything short of a full indoor ban on smoking may have no benefits and may, in fact, result in increased urges to smoke overall.
